# Supplementary material for: Reconciling Mining with the Conservation of Cave Biodiversity: A Quantitative Baseline to Help Establish Conservation Priorities
Source: PLoS One. 2016 Dec 20;11(12):e0168348. doi: 10.1371/journal.pone.0168348 (PMC5173368; doi:10.1371/journal.pone.0168348)
Supplement: S1 Dataset — (ZIP) [file pone.0168348.s002.zip › Taxa/Serra Norte/SN_2013/N5S/Lista N5S-21.pdf]

# CAVIDADE N5S-0021/0027

| Classe | Ordem | Fam/Outros | Gên/Outros | Espécie | S-U | U-S | Zon. |
|---|---|---|---|---|---|---|---|
| Annelida | Oligochaeta | | | jovem | 1 | | A |
| Arachnida | Acari | (Parasitengonina) | | sp.2 | X | | E |
| Arachnida | Acari | Bdellidae | | sp.61 | X | | P/A |
| Arachnida | Acari | Cunaxidae | | sp.1 | X | | A |
| Arachnida | Acari | Eupodidae | | sp.1 | X | | P/A |
| Arachnida | Acari | Ixodidae | | jovem | X | | A |
| Arachnida | Acari | Rhagidiidae | | sp.20 | X | | A |
| Arachnida | Acari | Trombiculidae | | sp.39 | X | | P |
| Arachnida | Acari | Trombidiformes | | sp.49 | X | | A |
| Arachnida | Acari | Trombidiidae | | sp.15 | X | | P/A |
| Arachnida | Acari | | | jovem | X | X | A |
| Arachnida | Amblypygi | Charinidae | *Charinus* | sp.1 | 1 | | A |
| Arachnida | Amblypygi | Phrynidae | *Heterophrynus* | *longicornis* | 2 | X | |
| Arachnida | Araneae | Caponiidae | | sp.1 | X | X | P/A |
| Arachnida | Araneae | Ochyroceratidae | *Ochyrocera* | sp.1 | X | X | E/P/A |
| Arachnida | Araneae | Ochyroceratidae | *Speocera* | sp.1 | X | | A |
| Arachnida | Araneae | Pholcidae | Ninetinae | sp.1 | X | | E |
| Arachnida | Araneae | Prodidomidae | *Lygromma* | sp.1 | X | | P |
| Arachnida | Araneae | Scytodidae | *Scytodes* | sp.1 | 5 | X | P |
| Arachnida | Araneae | Tetrablemmidae | *Matta* | sp.1 | X | | A |
| Arachnida | Araneae | Theridiosomatidae | *Plato* | sp.1 | X | X | E/P/A |
| Arachnida | Opiliones | Escadabiidae | | sp.2 | X | | P/A |
| Arachnida | Opiliones | Stygnidae | *Paraphareus* | sp.1 | X | | P |
| Arachnida | Palpigradi | Eukoeneniidae | *Allokoenenia* | sp.1 | X | | A |
| Arachnida | Pseudoscorpiones | Bochicidae | | sp.1 | X | | P |
| Arachnida | Pseudoscorpiones | Chthoniidae | *Pseudochthonius* | sp.2 | | X | |
| Arachnida | Pseudoscorpiones | Chthoniidae | | jovem | X | | A |
| Chilopoda | Geophilomorpha | Geophilidae | | sp.1 | 1 | | P |
| Chilopoda | Scolopendromorpha | Cryptopidae | *Cryptops* | jovem | 1 | | P |
| Diplopoda | Polydesmida | Fuhrmannodesmidae | | jovem | X | | A |
| Diplopoda | Polydesmida | Pyrgodesmidae | | sp.1 | X | X | P/A |
| Diplopoda | Siphonophorida | Siphonophoridae | | jovem | X | | P |
| Entognatha | Collembola | Cyphoderidae | | sp.1 | X | | P/A |
| Entognatha | Collembola | Entomobryidae | | sp.4 | X | | A |
| Entognatha | Collembola | Isotomidae | | sp.1 | X | | P/A |
| Entognatha | Collembola | Paronellidae | | sp.1 | X | | P |
| Entognatha | Collembola | Paronellidae | | sp.4 | X | | A |
| Entognatha | Collembola | Sminthuroidea | | sp.1 | X | | A |
| Entognatha | Collembola | Sminthuroidea | | sp.2 | X | | P/A |
| Entognatha | Diplura | Campodeidae | | sp.1 | X | | P |
| Entognatha | Diplura | Japygidae | | sp.1 | | X | |
| Insecta | Blattodea | Polyphagidae | | sp.1 | 6 | | E |
| Insecta | Coleoptera | Carabidae | *Coarazuphium* | sp.n.1 | X | X | P/A |
| Insecta | Coleoptera | Carabidae | *Gen.n.* | sp.17 | | X | A |
| Insecta | Coleoptera | Curculionidae | | sp. | | X | |
| Insecta | Coleoptera | Staphylinidae | | sp.10 | X | | A |
| Insecta | Diptera | Cecidomyiidae | Cecidomyiinae | sp. | X | | E |
| Insecta | Diptera | Culicidae | *Culex* | sp. | X | | E/A |
| Insecta | Diptera | Nematocera | | sp. | | X | |

| | | | | | | | |
|---|---|---|---|---|---|---|---|
| Insecta | Diptera | Phoridae | Phorinae | sp. | X | | P/A |
| Insecta | Diptera | Psychodidae | *Pifanomyia* | *gruta* | X | X | |
| Insecta | Heteroptera | Dipsocoroidea | | sp. | X | | P |
| Insecta | Heteroptera | Reduviidae | Emesinae | sp. | | X | |
| Insecta | Heteroptera | Reduviidae | Reduviinae | sp. | 1 | | P |
| Insecta | Homoptera | Cixiidae | | jovem | X | X | P |
| Insecta | Hymenoptera | Bethylidae | | sp. | | X | |
| Insecta | Hymenoptera | Formicidae | *Brachymyrmex* | sp.1 | X | | P |
| Insecta | Hymenoptera | Formicidae | *Dolichoderus* | aff. *bispinosus* | X | | E |
| Insecta | Hymenoptera | Formicidae | *Eurhopalothrix* | sp.1 | X | | P |
| Insecta | Hymenoptera | Formicidae | *Gnamptogenys* | sp.1 | X | X | P |
| Insecta | Hymenoptera | Formicidae | *Hypoponera* | sp.1 | X | | P/A |
| Insecta | Hymenoptera | Formicidae | *Labidus* | sp.1 | X | | A |
| Insecta | Hymenoptera | Formicidae | Myrmicinae | sp.1 | X | | P |
| Insecta | Hymenoptera | Formicidae | *Odontomachus* | sp. | | X | |
| Insecta | Hymenoptera | Formicidae | *Pachycondyla* | sp.2 | X | | E |
| Insecta | Hymenoptera | Formicidae | *Pheidole* | sp.2 | X | | P |
| Insecta | Hymenoptera | Formicidae | *Solenopsis* | sp.1 | X | | P/A |
| Insecta | Hymenoptera | Formicidae | *Trachymyrmex* | sp.1 | X | | P |
| Insecta | Isoptera | Termitidae | *Cornitermes* | sp. | X | | P |
| Insecta | Isoptera | Termitidae | *Embiratermes* | sp. | X | | P |
| Insecta | Isoptera | Termitidae | *Nasutitermes* | sp. | X | | E |
| Insecta | Lepidoptera | Tineoidea | | sp.1 | X | X | P |
| Insecta | Orthoptera | Phalangopsidae | *Paraclodes* | sp. | | X | E |
| Insecta | Orthoptera | Phalangopsidae | *Phalangopsis* | sp. | 89 | X | P |
| Insecta | Psocoptera | Epipsocidae | *Epipsocus* | sp.2 | X | X | P |
| Insecta | Thysanura | Ateluridae | | sp.1 | X | | P/A |
| Insecta | Thysanura | Nicoletiidae | | jovem | X | | E |
| Gastropoda | Pulmonata | Subulinidae | | sp.5 | X | | E |
| Malacostraca | Isopoda | Philosciidae | | sp. | | X | |
| Malacostraca | Isopoda | Scleropactidae | *Circoniscus* | *buckupi* | X | X | P/A |
| Symphyla | | Scutigerellidae | | sp. | X | | P |
| Amphibia | Anura | Leptodactylidae | *Eleutherodactylus* | cf. *fenestratus* | | X | |
| Mammalia | Chiroptera | Emballonuridae | *Peropteryx* | sp. | | X | |
